# Oyster Peptides Prepared by *Lactobacillus casei* Fermentation Enhance Immune Activity in RAW264.7 Cells via Activation of the MAPK Pathway

**DOI:** 10.3390/md23120484

**Published:** 2025-12-18

**Authors:** Lingyue Zhong, Yirui Wu, Xuefang Guan, Mei Xu, Juqing Huang, Yafeng Zheng, Qi Wang

**Affiliations:** 1 Institute of Food Science and Technology, Fujian Academy of Agricultural Sciences, Fuzhou 350003, China; lingyue_zhong@163.com (L.Z.); guan-619@163.com (X.G.); jq_huang@zju.edu.cn (J.H.); 2 Yuanzhidao-FAAS Joint Innovation Center for Functional Probiotics & Medicinal-Edible Resources, Xiamen 361199, China; 18259288898@163.com; 3 College of Food Science, Fujian Agriculture and Forestry University, Fuzhou 350002, China; wyr516999@163.com; 4 Fujian Key Laboratory of Agricultural Product (Food) Processing, Fuzhou 350003, China

**Keywords:** oyster, peptide, microbial fermentation, immunomodulatory activity

## Abstract

Oyster peptides (OPs) have gained increasing attention for their excellent biological activities, especially immunomodulatory effects. In this study, oyster proteins were fermented using *Lactobacillus casei* to prepare bioactive peptides, and the effects of fermentation parameters (time, temperature, and inoculum amount) on the degree of hydrolysis (DH) were optimized. The optimal fermentation conditions were determined as 30 h, 35 °C, and 5% inoculum amount, resulting in a DH of 28.24%. Structural characterization showed that OPs were mainly composed of low-molecular-weight peptides (<1000 Da) with high hydrophobic amino acid content, and they exhibited good stability during in vitro gastrointestinal digestion. In vitro immunological evaluation using RAW264.7 macrophages demonstrated that OPs significantly enhanced phagocytic activity and nitric oxide (NO) production, and upregulated the mRNA expression levels of pro-inflammatory cytokines including interleukin (IL)-6, IL-1β, and tumor necrosis factor (TNF)-α. Mechanistically, OPs exerted immunostimulatory effects by specifically activating the extracellular signal-regulated kinase (ERK) pathway within the mitogen-activated protein kinase (MAPK) signaling cascade, without significant alterations in the phosphorylation levels of p38 and c-Jun N-terminal kinase (JNK). These findings highlight the potential of *Lactobacillus casei*-fermented oyster peptides as natural immunomodulatory ingredients for functional food development.

## 1. Introduction

Oysters, warm-water bivalve mollusks inhabiting marine ecosystems, are a valuable source of high-quality protein, with protein contents ranging from 39.1% to 53.1% [1]. Furthermore, oysters contain bioactive components such as amino acids, essential vitamins, minerals, taurine, polysaccharides, eicosapentaenoic acid (EPA), and docosahexaenoic acid (DHA), which have potential health benefits such as antioxidant, immune regulation, and anti-fatigue effects [2,3]. Against the backdrop of the continuous expansion of global marine resource development, oysters—an important marine shellfish with inherent functional food properties—stand out for their high nutritional value, prominent health-promoting effects, and substantial application potential in functional nutrition. This intrinsic functional attribute endows them with broad prospects for development in the field of nutritional and functional food production. In recent years, there has been an annual increase in oyster production. According to relevant data, the production of oysters in China’s marine aquaculture reached 5.8192 million tons in 2022 and 6.1995 million tons in 2023, representing a year-on-year increase of 6.53% [4]. Establishing a high-efficiency oyster processing system underpinned by advanced biotechnology is essential, as it tackles two core industry challenges. First, China’s oyster aquaculture output has maintained steady growth, yet most products are low-value primary goods plagued by short shelf lives and limited added value. Second, inefficient processing practices result in underutilization of oyster resources and associated waste. Shifting from conventional primary products to high-value-added derivatives is therefore critical to advancing resource optimization and sustainable industrial development. This transformation is of paramount importance in promoting the effective utilization of marine biological resources and ensuring the sustainable development of the industry.

Oysters have emerged as a valuable source of raw materials for the development of functional peptides. A substantial body of research has validated the anti-inflammatory and immune-boosting properties of oyster enzymatic hydrolysis products and active peptides isolated from these mollusks. Hwang et al. identified a variety of bioactive peptide segments derived from diverse oyster varieties. Specifically, peptide segments derived from oysters have been shown to effectively inhibit lipopolysaccharide-induced activation of human macrophages (RAW264.7) and nitric oxide production [5]. Another study discovered that β-thymosin, a protein isolated from water oysters, can modulate the NF-κB signaling pathway, impede the production of inflammatory factors such as PGE2, and diminish the expression levels of iNOS and COX-2 [6]. Furthermore, it has been demonstrated that oyster peptides can mitigate liver damage through a variety of mechanisms, including the suppression of inflammatory responses and cell apoptosis processes [7]. Li et al. found that this component not only promotes lymphocyte proliferation, but also enhances the phagocytic activity and NO production ability of macrophages. Subsequent separation and purification techniques were employed to identify two characteristic peptide segments with immune regulatory functions, whose amino acid sequences are DNSIAMESMK and LLQLSER [8].

Microbial fermentation represents a pivotal technology for the preparation of bioactive peptides. This process entails the degradation of protein substrates and the subsequent generation of active peptides with specific functions through the secretion of proteases or peptidases by designated microorganisms (e.g., lactic acid bacteria, Bacillus subtilis, and yeast) during the fermentation process [9,10]. The fundamental mechanism entails the breakdown of substrate proteins by microorganisms through their own metabolic activities, accompanied by the generation of secondary metabolites. This process may result in diversity in the functional properties of active peptides due to differences in bacterial characteristics, fermentation conditions, and substrate sources. In comparison with alternative preparation methodologies, the economic expense of producing bioactive peptides by microbial fermentation is notably reduced [11]. Furthermore, during the process of forming bioactive peptides, it has been observed to enhance the texture and flavor of the final product [10].

Presently, studies have applied microbial fermentation to the production of oyster active peptides. Lee et al. found that the fermentation product obtained from oyster fermented by *Lactobacillus brevis* promoted the growth and development of rats and improved their bone mineral density [12]. Lhn et al. found that the oyster peptide obtained from *Lactobacillus brevis* BJ20 fermentation prevented ovariectomy-induced bone loss [13]. However, oyster peptides prepared by *Lactobacillus casei* fermentation and their immunomodulatory effects have not yet been reported. *Lactobacillus casei* was chosen because it not only exhibits documented immunomodulatory activity via the strain and its metabolites [14] but also demonstrates high oyster protein degradation efficiency according to our preliminary studies. The present study has thus been devised to prepare oyster peptides through microbial fermentation, evaluating their immune activity based on cellular immune models, and exploring their mechanisms of action. The results will provide a theoretical basis for optimizing the microbial fermentation process for preparing active oyster peptides and verifying their functional activities, thereby laying a foundation for the high-value utilization of oysters and the in-depth research and development of oyster peptide products.

## 2. Results

### 2.1. Optimization of Oyster Homogenate Fermentation Parameters

#### 2.1.1. Single-Factor Analysis

The fermentation of microbes has been identified as a novel method for the production of bioactive peptides, with mild conditions and a rich variety of hydrolyzed products being key features. The efficiency of preparing active peptides is affected by a variety of factors, including fermentation time, temperature, pH, and strain concentration. In the present study, the effects of inoculum amount, fermentation time, and temperature on the hydrolysis degree of oyster protein were investigated in conjunction with preliminary experiments.

**Figure 1:**
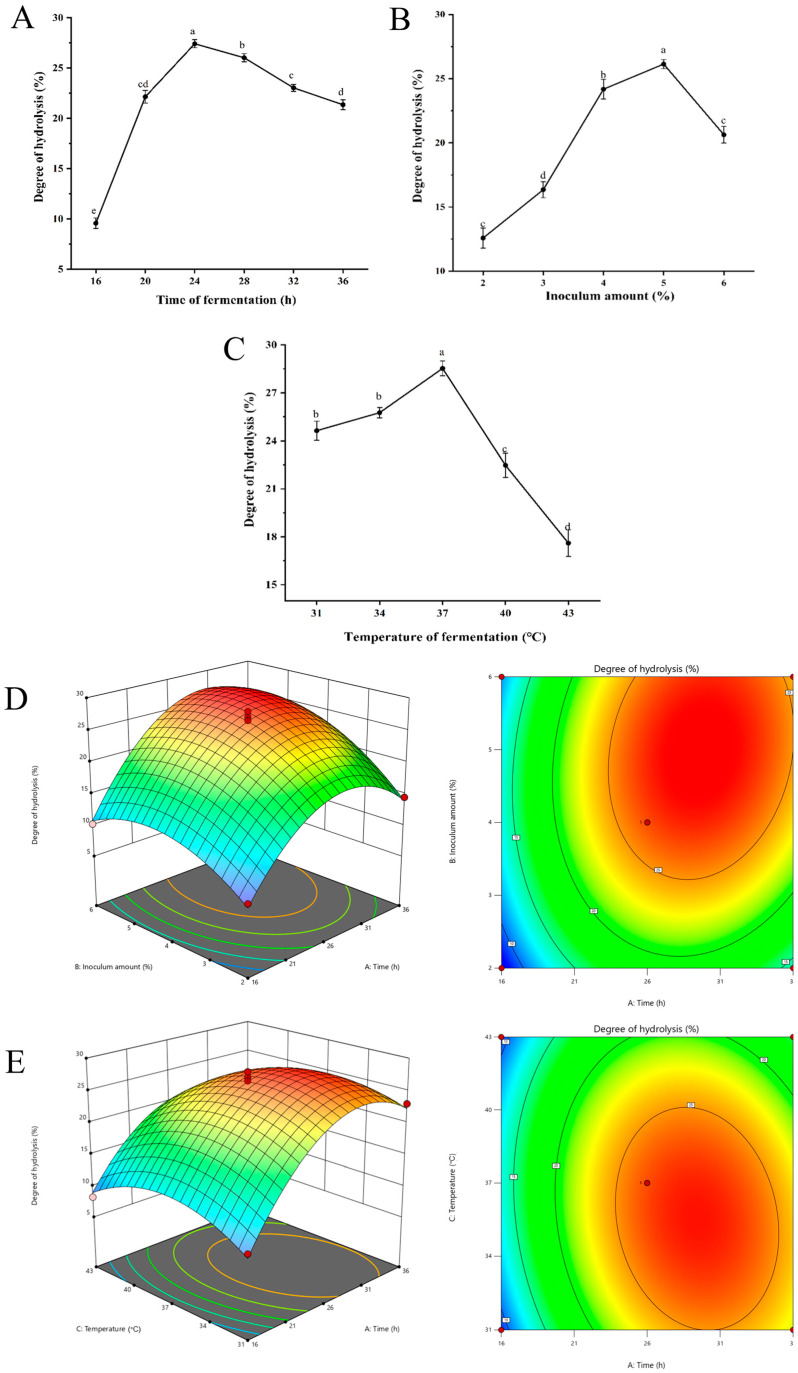
Optimization of oyster homogenate fermentation parameters to obtain the highest degree of hydrolysis, (**A**–**C**) Effect of fermentation time (**A**), inoculum amount (**B**), and fermentation temperature (**C**) on the degree of hydrolysis (%) of oyster peptides. (**D**,**E**) Response surface methodology analysis showing interactive effects between (**D**) fermentation time and inoculum amount, and (**E**) fermentation time and temperature; both 3D surface and 2D contour plots were constructed using Design-Expert 13. Different letters above bars indicate significant differences (*p* < 0.05) among treatment parameters.

As illustrated in Figure 1A, the degree of hydrolysis increased when the fermentation time extended from 16 to 24 h. The degree of hydrolysis significantly increased to 27.41% when the fermentation time was 24 h, compared to 16 h fermentation (*p* < 0.05). For the inoculum amount of *Lactobacillus casei*, as shown in Figure 1B, the hydrolysis degree of oyster protein increased significantly with the increasing inoculum amount in the range of 2–5% inoculum (*p* < 0.05). The maximum degree of hydrolysis (26.14%) was achieved at an inoculum amount of 5%. As demonstrated in Figure 1C, the degree of hydrolysis exhibited a gradual increase in response to elevated temperatures, ranging from 31 °C to 37 °C. At a fermentation temperature of 37 °C, the degree of hydrolysis was recorded to be 28.53%. Conversely, the hydrolysis degree exhibited a rapid decrease as the temperature continued to rise to 43 °C.

#### 2.1.2. Response Surface Test

The results of the response surface test designed using the Box–Behnken method are presented in Table S3. The regression model was fitted by Design-Expert 13 software. The multiple quadratic regression simulation equations between the degree of hydrolysis of oyster protein (Y) and the three factors of fermentation time (A), inoculum amount (B), and temperature (C) were as follows: Y = 26.41 + 5.11 × A + 3.48 × B − 1.56 × B − 1.563.48 × B − 1.56 × C + 1.72 × AB − 1.63 × AC − 0.53 × BC − 8.04 × A^2^ − 4.26 × B^2^ − 4.37 × C^2^.

The analysis of variance is shown in Table S4. The regression model *p* < 0.0001 and misfit term *p* = 0.3707 > 0.05, indicating that the model was well fitted. The adjusted regression coefficient R^2^ = 0.9655, indicating that 96.55% of the data could be interpreted by the model. The relative importance of individual factors quantified by F-values was: fermentation time (A) > inoculum (B) > temperature (C). The secondary term interactions AB and AC exerted a significant effect on the degree of hydrolysis (*p* < 0.05), which was consistent with the steepness of the surface slope observed in the response surface plots (Figure 1D,E). The results indicated that the interaction between fermentation time (A) and inoculum amount (B), as well as fermentation time (A) and temperature (C), exhibited a high degree of significance.

The software analysis showed that the optimal condition for the fermentation of oyster protein hydrolysis was as follows: time of 30.013 h, inoculum amount of 5.015%, and temperature of 35.292 °C. According to the actual conditions of the experiment, the conditions were modified as follows: time of 30.0 h, inoculum amount of 5.0%, and temperature of 35 °C. The degree of hydrolysis was verified to be 28.244 ± 1.127% under optimal conditions. The relative standard deviation (RSD) was 1.02% compared to the predicted value (28.535%), indicating that the predicted and experimental values were in good agreement.

### 2.2. Characterization of Oyster Peptide Structure

#### 2.2.1. Full-Wavelength UV Scanning

**Figure 2:**
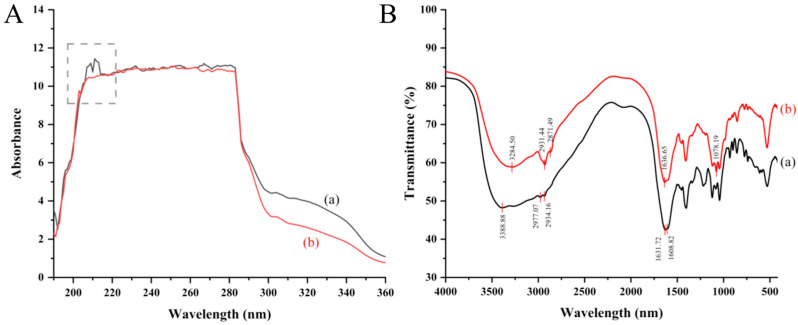
Spectral characterization of oyster peptide structure alternation before (OP) and after (DOP) simulated digestion. (**A**) Full-wavelength ultraviolet (UV) scanning of OP (a) and DOP (b). The characteristic peak of OP observed at around 210 nm was marked with dashed lines, which is due to the peptide bonding. (**B**) Fourier transform infrared spectrum of OP (a) and DOP (b). The characteristic absorption peaks for OP (a) appeared at 3388.88 cm^−1^, 2977.07 cm^−1^, 2934.16 cm^−1^, 1631.72 cm^−1^, and 1608.82 cm^−1^, and for DOP (b) appeared at 3284.50 cm^−1^, 2931.44 cm^−1^, 2871.49 cm^−1^, 1636.65 cm^−1^, and 1078.19 cm^−1^.

The results of full-wavelength UV scanning of OP (a) and DOP (b) are shown in Figure 2A. The characteristic peak of peptide bonding was observed at 210 nm, which was due to the π→π* leaps and n→π* leaps, indicating that several peptide bonds existed in OP [15]. Another characteristic peak of peptide around 270 nm was due to the π→π* leaps of the aromatic ring structure of the amino acid, such as tyrosine, tryptophan, and phenylalanine [16]. The results showed the low absorption intensity of OP at 270 nm, indicating that OP contained a minimal amount of aromatic amino acids. After digestion by a simulated gastrointestinal system, the characteristic peak at 200–220 nm disappeared, indicating that OP was partially hydrolyzed to the small peptides and amino acids during simulated gastrointestinal digestion, which may be beneficial for the absorption by small intestinal epithelial cells.

#### 2.2.2. FTIR Spectral Analysis

The scanned results of the FTIR spectra of OP (a) and DOP (b) in the range of 4000–500 cm^−1^ are shown in Figure 2B. The characteristic absorption peaks for OP appeared at 3388.88 cm^−1^, 2977.07/2934.16 cm^−1^, 1631.72 cm^−1^, and 1608.82 cm^−1^, corresponding to the stretching vibrations of N-H bonds, C-H bonds, C=O bonds, and the amide I band, respectively. The intense absorption in the amide I spectral region of OP indicated the presence of amino acid side chains of globular proteins (e.g., Arg, Glu, Asp) [17].

Similarly, the characteristic absorption peaks for DOP appeared at 3284.50 cm^−1^, 2931.44/2871.49 cm^−1^, and 1636.65 cm^−1^, corresponding to the stretching vibrations of N–H bonds, C–H bonds, C=O bonds, and the amide I band, respectively. The FTIR spectra of DOP showed similar characteristic peaks to OP, suggesting that the core polypeptide backbone remains intact after simulated digestion. While this structural preservation suggests potential for retained bioactivity, functional activity must be confirmed through biological assays, which we demonstrated subsequently using RAW264.7 cells.

#### 2.2.3. Total and Free Amino Acid Composition

The total amino acid composition and content of OP and DOP were summarized in Table 1. Results showed that the total amino acid content of OP was 144.7 mg/g, among which the essential amino acids accounted for 24.81% and the hydrophobic amino acids accounted for 32.44%. The major amino acids of OP were Gly (18.96%), Glu (16.61%), Ala (10.78%), Asp (9.77%), and Pro (7.28%). The total amino acid content of DOP was 141.98 mg/g, among which essential amino acids accounted for 29.73% and hydrophobic amino acids accounted for 31.13%. The major amino acids of DOP were Glu (15.62%), Gly (12.42%), Asp (12.30%), Ala (7.89%), and Pro (5.49%). The results indicated that the amino acid composition of OP remained relatively stable during the digestion process.

The free amino acid composition of OP and DOP is shown in Table 2. The total free amino acid content of OP was 134.77 mg/g, among which the essential amino acids accounted for 28.86% and the hydrophobic amino acids accounted for 29.67%. The total free amino acid content of DOP was 141.58 mg/g, among which the essential amino acids accounted for 42.09% and the hydrophobic amino acids accounted for 34.23%. The results indicated that the contents of essential amino acids and hydrophobic amino acids in the free amino acids of OP were increased after digestion.

#### 2.2.4. Relative Molecular Mass Distribution

**Figure 3:**
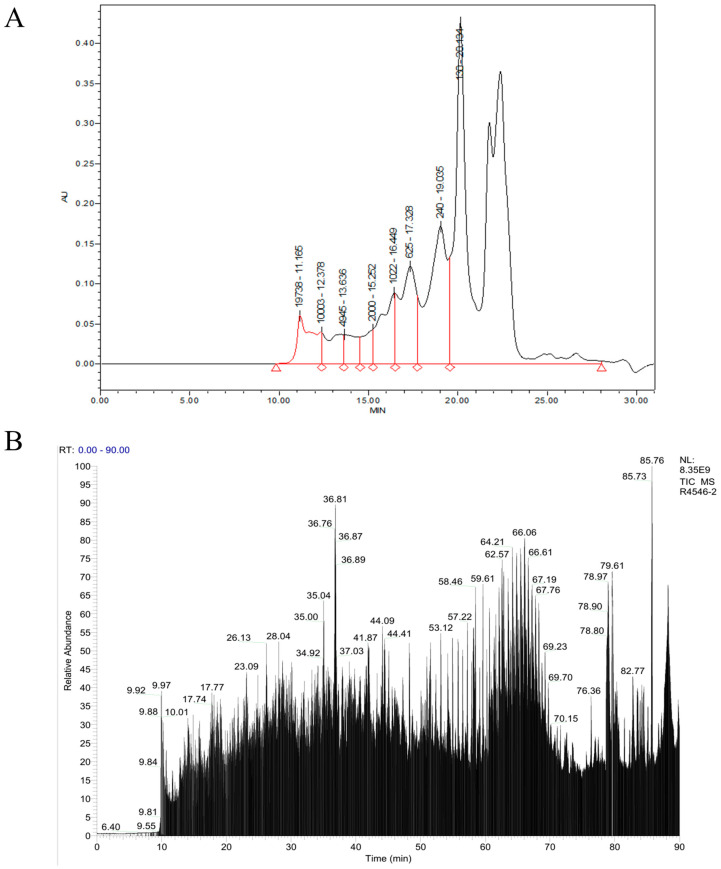
Molecular characterization of oyster peptide (OP). (**A**) RP-HPLC chromatogram showing molecular mass distribution, with masses indicated above respective peaks. (**B**) LC-MS/MS total ion chromatogram used for peptide sequence identification *via* De novo sequencing.

As demonstrated in Figure 3A and Table 3, the components with molecular weights below 1000 Da accounted for 82.11% of the total OP components, and the interval of less than 180 Da accounted for as high as 57.46%, indicating that the OP was mainly composed of small-molecule peptides.

#### 2.2.5. LC-MS/MS Peptide Sequence Identification

Peptide sequence identification of OP was performed by LC-MS/MS and the De novo method, and the total ion flow diagram of OP is shown in Figure 3B. Research has demonstrated that active peptides with immunomodulatory functions typically comprise 2–20 amino acids, with a preponderance of hydrophobic amino acids [18,19]. Consequently, an integration of the characteristics of immunoreactive peptides and the PeptideRanker algorithm score was employed for the purpose of filtering 20 peptides with potential immunomodulatory functions, as illustrated in Table 4. The relative molecular weights of these peptides are principally distributed in the range of 400–1700 Da, and all of them have hydrophobic amino acids, which can interact with cell membrane proteins and thus improve the structural stability of the molecules.

### 2.3. Effects of OP on Immunoreactivity of RAW264.7 Cell Lines

#### 2.3.1. Effects of OP on the Morphology, Viability, and Phagocytosis of RAW264.7 Cell Lines

**Figure 4:**
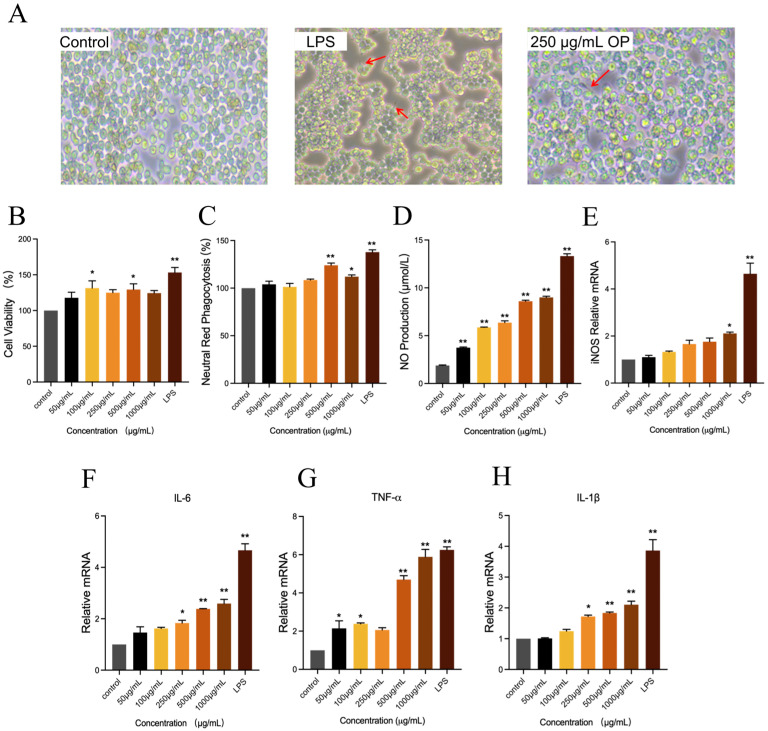
The activation effects of oyster peptide (OP) on the immune activity of RAW264.7 cell lines. (**A**) The morphology alternation of RAW264.7 cells in control, LPS, and 250 μg/mL OP treatment groups was observed under the microscope. The emergence of pseudopods of RAW264.7 cells was marked by red arrows. (**B**) Cell viability of RAW264.7 in different groups, detected by CCK-8 kits. (**C**) Effects of OP on the proliferation rate of RAW264.7 cells in different groups, represented by neutral red phagocytosis. (**D**) NO concentration of the culture supernatants of RAW264.7 cells in different groups. (**E**–**H**) The relative mRNA expression of iNOS (**E**), IL-6 (**F**), TNF-α (**G**), and IL-1β (**H**) of RAW264.7 cells in different groups. * *p* < 0.05, ** *p* < 0.01 vs. the control group.

Macrophage activation was frequently accompanied by alterations in cell morphology. As demonstrated in Figure 4A, the typical appearance of RAW264.7 cells was characterized by a near-oval shape and a smooth surface in the control group. In the LPS group, RAW264.7 cells demonstrated notable differentiation, manifesting an increased number of cells with pseudopods and a dendritic morphology. In comparison with the control group, the application of 250 μg/mL of OP resulted in an augmentation of cell differentiation, accompanied by the emergence of pseudopods (indicated by red arrows). This observation served as an indication that OP exerted an activating effect on RAW264.7 cells.

The effects of different concentrations of oyster peptide on RAW264.7 cell viability were detected by CCK-8 assay. As shown in Figure 4B, compared with the control group, the OP intervention of 50–1000 μg/mL resulted in a certain degree of elevation of cell viability, and the cell viability was significantly increased at the intervention concentrations of 100 μg/mL and 500 μg/mL (*p* < 0.05). The findings of the study indicated that the OP enhanced cell viability within a certain concentration range.

Phagocytosis is a process through which cells engulf microbial pathogens, representing a pivotal function of macrophages [20]. As demonstrated in Figure 4C, the phagocytosis of the cells was significantly increased when the OP concentration was 500 μg/mL and 1000 μg/mL in comparison with the control group (*p* < 0.05).

#### 2.3.2. OP Improved NO Secretion in RAW264.7 Cell Lines

NO has been identified as the primary effector molecule of macrophages, playing a pivotal role in defense against pathogens, regulation of immune cell function, and immune metabolism [21]. Therefore, NO secretion of cells in each treatment was detected (Figure 4D). Following the administration of OP at varying concentrations, a significant increase in NO production was observed in RAW264.7 cells in comparison with the control group (*p* < 0.01). This response exhibited a concentration-dependent relationship, indicating that the magnitude of the effect increased with increasing concentrations of oyster peptide.

iNOS represents a significant class of enzymes that play a pivotal role in the synthesis of NO within the body [22]. In order to investigate the mechanism of OP enhanced NO secretion in RAW264.7 cells, iNOS mRNA gene expression was examined further (Figure 4E). OP elevated iNOS mRNA expression levels in cells compared to the control group with a concentration-dependent change. The 1000 μg/mL concentration OP treatment group has been observed to significantly up-regulate the expression level of iNOS mRNA (*p* < 0.05).

#### 2.3.3. OP Improved IL-6, IL-1β and TNF-α mRNA Expression Levels in RAW264.7 Cell Lines

The production of IL-6 is primarily undertaken by a variety of cells, including macrophages, T cells, and B cells. It has been demonstrated to regulate the growth and differentiation of a variety of cells, to possess the ability to regulate immune response, acute phase response, and haematopoietic function, and to play an important role in the anti-infection immune response [23]. As demonstrated in Figure 4F, the mRNA expression level of IL-6 in the LPS group was significantly higher than that in all other treatment groups (*p* < 0.01). Furthermore, IL-6 mRNA expression in the OP-treated groups exhibited a dose-dependent relationship: the 250 μg/mL group showed a significant up-regulation compared to the control group (*p* < 0.05), while the high-concentration groups (500 μg/mL and 1000 μg/mL) displayed a more pronounced elevation in IL-6 mRNA expression (*p* < 0.01).

TNF-α, a small-molecule protein secreted by macrophages, is one of the bioactive factors with the strongest direct tumor-killing effect [24]. As shown in Figure 4G, TNF-α mRNA expression was significantly increased in the 250 μg/mL OP group compared to the control (*p* < 0.05), with further pronounced elevation in the 500 μg/mL and 1000 μg/mL groups (*p* < 0.01).

IL-1β is a cytokine produced by activated macrophages, which stimulates proliferation, differentiation, and improves the function of cells involved in the immune response [25]. For IL-1β mRNA (Figure 4H), OP treatment induced a concentration-dependent upregulation: the 250 μg/mL group exhibited a significant increase (*p* < 0.05), while the 500 μg/mL and 1000 μg/mL groups showed highly significant differences (*p* < 0.01).

#### 2.3.4. OP Activated the MAPK Signaling Pathway in RAW264.7 Cell Lines

**Figure 5:**
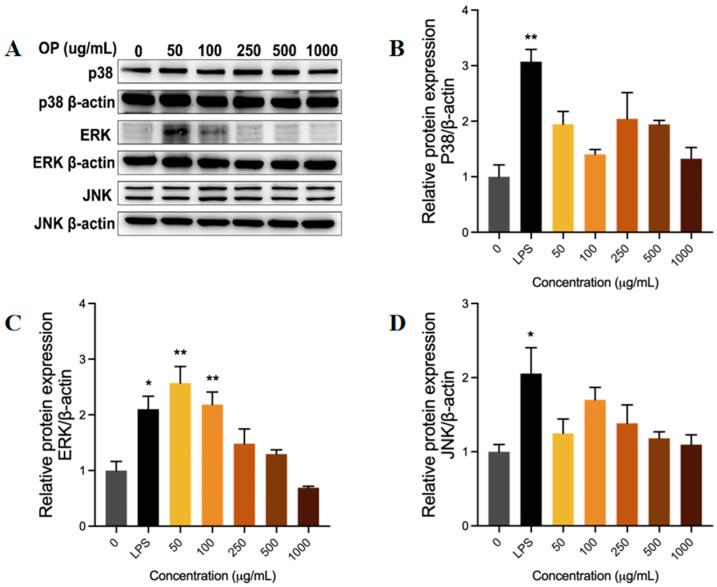
The effect of oyster peptide (OP) on the activation of the MAPK signaling pathway in RAW264.7 cell lines. (**A**) Protein phosphorylation levels detected by Western blot. (**B**–**D**) The relative phosphorylation level of p38 (**B**), ERK (**C**), and JNK (**D**) proteins in different groups. * *p* < 0.05, ** *p* < 0.01 vs. the control group.

The present study further investigated the relationship between different concentrations of OP and the phosphorylation levels of P38 (Figure 5B), ERK (Figure 5C), and JNK (Figure 5D) in the MAPK signaling pathway by Western blot (Figure 5A). This approach was adopted as a means of assessing the effect of OP on the MAPK signaling pathway.

LPS stimulation led to a significant increase (*p* < 0.05) in the phosphorylation levels of P38, ERK, and JNK proteins in RAW246.7 cells compared to the control group, thereby inducing a substantial activation of the MAPK signaling pathway. The levels of p38, ERK, and JNK phosphorylation in macrophages cultured with low concentrations of OP were higher than those in the control group. Notably, ERK phosphorylation was selectively increased at low OP concentrations (50–250 μg/mL) compared to the control group (*p* < 0.01), while p38 and JNK showed no significant difference compared to the control group. The selective activation of ERK is likely to mediate MAPK pathway activation, a mechanism that may underlie the immunostimulatory effects observed in RAW264.7 cells.

## 3. Discussion

In this study, we prepared oyster peptides by *Lactobacillus casei* fermentation and further detected the structure, digestive stability, and immune activation of the peptides. A study was conducted to examine the impact of key fermentation parameters, including duration, temperature, and inoculum amount, on the degree of hydrolysis of OP. The findings indicated that with an increase in fermentation time, temperature, and inoculation amount, there was a tendency for the hydrolysis degree to initially rise and subsequently decline. The fundamental principle underlying the production of bioactive peptides through microbial fermentation entails the breakdown of proteins by proteases, which are enzymes secreted by microorganisms during their growth process [26]. Consequently, the extent of protein hydrolysis is directly proportional to the level and activity of proteases secreted by microorganisms. Excessively long fermentation duration, oversized inoculum, and improper temperature can inhibit microbial growth or protease activity, consequently adversely affecting the degree of hydrolysis [27].

The relative molecular weight analysis of OPs revealed that they are predominantly composed of small-molecule (<1000 Da) peptide segments. Research has shown that small-molecule active peptides possess a straightforward structure and robust biological activity, while also exhibiting substantial stability and safety advantages [28]. These peptides have the capacity to traverse the intestinal barrier and be rapidly and residue-free absorbed into the body without further decomposition. The rapid absorption of low-molecular-weight peptides has been demonstrated to facilitate the establishment of a more balanced amino acid pattern in the blood, a process that is imperative for the maintenance of physiological balance within the body [29].

With respect to the total amino acid composition of OP, a high proportion of hydrophobic amino acids was observed, with Gly being the most abundant among all amino acids. Studies have shown that hydrophobic amino acids are the characteristic amino acids of immunomodulatory peptides [30]. In addition, peptides containing Gly, Tyr, and Pro exhibited better immunomodulatory activity [18]. Furthermore, the content of essential and hydrophobic amino acids in the free amino acids of OP increased after digestion, with a notable increase in the content of Arg. Arg has been demonstrated to participate in the body’s metabolic processes and promote immune cell proliferation [31].

The immunomodulatory function of OP was evaluated using RAW264.7 cells. The results demonstrated that OP promoted the differentiation of RAW264.7 cells and enhanced their phagocytic activity. A salient finding of the study was the observation that OP significantly upregulated the mRNA expression levels of immune-related cytokines, including IL-6, IL-1β, and TNF-α, in RAW264.7 cells, which suggests a potential tendency toward enhanced immune-associated transcriptional activity in these cells. Previous studies have also reported that peptides stimulate the immunomodulatory function in cells and the body. Shao et al. revealed that ovalbumin peptide alleviated cyclophosphamide (CTX)-induced immune dysfunction of mice by enhancing the secretion of immunoglobulins, IL-2, IL-6, and TNF-α [32]. Another study utilized shrimp peptide to stimulate RAW264.7 cells. The results demonstrated that the spreading and pseudopodia formation of the cells underwent a gradual elongation, and the capability of non-specific immunity and phagocytosis was enhanced under peptide intervention [33].

Compared with previous studies on oyster peptides, our fermentation-derived OP exhibits distinct characteristics in both peptide composition and biological activity. While enzymatic hydrolysis studies identified peptides with immunoregulatory functions [8], and others reported anti-inflammatory peptides that inhibit NO production and suppress NF-κB signaling [5,6], our OP preparation contains novel peptide sequences (Table 4) with pronounced immunostimulatory effects. This divergence can be mechanistically attributed to the unique proteolytic system of *Lactobacillus casei*, which generates different cleavage patterns. Furthermore, fermentation introduces bacterial cell wall components and metabolites that may synergistically activate macrophages through pattern recognition receptors, complementing the direct effects of the peptides themselves. This multifaceted activation likely explains why our OP preparation enhances NO production and pro-inflammatory cytokine expression through the MAPK pathway, contrasting with the NF-κB inhibition reported for some enzymatically derived oyster peptides [6]. The enrichment of hydrophobic amino acids and specific residues like Gly and Arg further distinguishes our peptide profile and contributes to its unique immunomodulatory mechanism.

Furthermore, the present study indicated that selective activation of the ERK pathway may contribute to MAPK pathway activation, which is correlated with the observed immunostimulatory effects of OP. It has been reported that the p38, JNK, and ERK MAPK signaling pathways are located upstream of IL-1β, IL-6, and TNF-α [34], and activation of the MAPK pathway is implicated in the regulation of immune responses to pathogens. Ren et al. found that peptides from monkfish roe activated the NF-κB and MAPK pathways in the spleen tissues and enhanced the serum levels of IL-6, IL-1β, and TNF-α on CTX-induced immunosuppressed mice [35]. In the context of the current study, these observations collectively suggest a potential correlation between OP-induced activation of the MAPK signaling pathway and the upregulated mRNA expression of immune-related cytokines. While this raises the possibility that the MAPK pathway may serve as a candidate mediator underlying OP’s immunomodulatory effects in RAW264.7 cells, the complex nature of cellular signaling—including regulatory crosstalk, negative feedback loops, and post-transcriptional/post-translational controls—means a direct or exclusive causal relationship cannot be definitively established. Thus, the MAPK pathway may represent a plausible potential mechanism through which OP could modulate immune-related transcriptional activity, but further studies are required to validate the functional relevance of this signaling cascade and its direct contribution to downstream immune outcomes.

## 4. Materials and Methods

### 4.1. Materials and Chemical Reagents

The oysters were sourced from the coastal waters of southeastern China (Fuzhou, China), and the species was identified as *Crassostrea angulata*. *Lactobacillus casei* (BNCC 186562) was purchased from BNCC Biotechnology Co., Ltd. (Beijing, China), following its screening as the target strain based on preliminary experimental results. Simulated gastric fluid and simulated intestinal fluid were purchased from Shanghai Yuanye Biotechnology Co., Ltd. (Shanghai, China). LPS was purchased from Biosharp (Beijing, China). Primary antibodies against p38, ERK, JNK, phosphorylated-p38 (p-p38), phosphorylated-ERK (p-ERK), phosphorylated-JNK (p-JNK), and β-actin, as well as corresponding secondary antibodies, were purchased from Sinopharm (Shanghai, China). All other reagents were procured from Sinopharm (Shanghai, China).

### 4.2. Preparation of Oyster Active Peptides

The preparation of oyster active peptides was conducted following the microbial fermentation protocol with minor modifications [13]. The oyster shells were detached to collect oyster tissue. Subsequently, distilled water was added to oyster tissue at a solid–liquid ratio of 1:3 (g/mL). The mixture was then subjected to intermittent homogenization at a speed of 8000 r/min for a duration of 30 s, with an interval of 10 s between each cycle, for a total of five repetitions. The homogenate was then deodorized with 0.1% activated carbon for 30 min, followed by sub-packing into a 250 mL conical flask. Subsequently, the pH was adjusted to 7, and the homogenate was autoclaved at 121 °C for 15 min. The homogenate was cooled, and the oyster fermentation medium was obtained.

*Lactobacillus casei* was inoculated anaerobically at 37 °C for 24 h with shaking at 180 r/min, and activated to three generations. The Box–Behnken experimental design (Table S1) was utilized to ascertain the fermentation treatment that exhibited the highest degree of hydrolysis. The single-factor experiments that were designed included the fermentation time (16, 20, 24, 28, 32, 36 h), inoculation volume of bacterial liquid (2, 3, 4, 5, 6 mL/100 mL), and fermentation temperature (31, 34, 37, 40, 43 °C). These parameter ranges were selected based on the typical adaptive tolerance and optimal growth conditions of *Lactobacillus casei* reported in previous studies [36,37], combined with preliminary experiments verifying their effectiveness for oyster protein degradation. The fermented mixture was subjected to a centrifugal process (9000 r/min, 4 °C, 20 min) and subsequently filtered using a 0.45 μm microporous filter membrane. The supernatant was then subjected to freeze-drying to obtain OPs and stored at a temperature of −20 °C.

### 4.3. Determination of the Degree of Hydrolysis

The degree of hydrolysis of the oyster peptide fermentation broth was determined by means of the trichloroacetic acid (TCA) method [38]. An equal volume of oyster peptide solution and 10% TCA was mixed and shaken, and then subjected to centrifugation at 4000 r/min for 20 min. The supernatant was then diluted, and the content of soluble polypeptides was measured before (N1) and after (N2) fermentation by means of the Folin phenol method. The total protein content (N0) was determined by means of the micro Kjeldahl method. The degree of hydrolysis (DH%) is calculated using the following formula: DH% = (N2 − N1)/(N0 − N1) × 100%.

### 4.4. Gastrointestinal Digestion Simulation

The in vitro gastrointestinal digestion simulation was carried out according to the reported procedure [39]. Simulated gastric juice (Cat. No. R28616, hydrochloric acid solution, pH = 2.0 ± 0.5, pepsin 1% w/v) and simulated intestinal juice (Cat. No. R22156, phosphate-buffered solution, pH = 6.8, trypsin concentration: 0.464 g/L) were procured from Yuanye Biotechnology Co., Ltd. (Shanghai, China). The pH value of the OP aqueous solution (mass concentration of 50 mg/mL) was adjusted to 2 using 1.0 M HCl. Following a preliminary heating step at 37 °C, an equal volume of simulated gastric juice was added. The mixture was subjected to a process of digestion, utilizing a constant temperature shaker maintained at 37 °C for a duration of three hours. Subsequently, the mixture was subjected to a boiling water bath for a period of ten minutes. The pH was adjusted to 6.8 with 1 mol/L NaOH, after which an equal volume of simulated intestinal fluid was added. The mixture was subjected to a process of digestion, utilizing a constant temperature shaker maintained at 37 °C for a duration of three hours. Subsequently, the mixture was transferred to a boiling water bath for a period of ten minutes. Following a period of cooling to ambient temperature, the pH was adjusted to a value of 7. The resultant mixture was then subjected to a process of centrifugation, after which the upper layer was removed. The product was freeze-dried to obtain the digested oyster peptide (DOP).

### 4.5. Full-Wavelength Ultraviolet (UV) Scanning

A 10 mg/mL oyster peptide solution was prepared with a PBS solution. The sample was then subjected to full-wavelength UV scanning in the spectral range of 190 nm to 410 nm using an ultraviolet spectrophotometer (nanodrop 2000C, Thermo Fisher, Waltham, MA, USA).

### 4.6. Fourier Transform Infrared (FTIR) Spectral Analysis

The oyster peptides were mixed with potassium bromide and thoroughly ground. A Fourier transform infrared spectrometer (Nicolet iS50, Thermo Fisher, Waltham, MA, USA) was utilized to measure the FTIR spectrum in the spectral range of 4000–500 cm^−1^.

### 4.7. Determination of Amino Acids

For total amino acids analysis, the samples were mixed with 6.0 M HCl and hydrolyzed at 110 °C for 24 h. For free amino acids analysis, the sample was thoroughly mixed with an equal volume of 5% (w/v) sulfosalicylic acid to precipitate the proteins. The supernatant was evaporated to dryness in a rotary evaporator, dissolved in sodium citrate buffer, and filtered through an aqueous 0.45 μm membrane. The composition of total and free amino acids was analyzed using a fully automated amino acid analyzer (L8080, Hitachi, Tokyo, Japan).

### 4.8. Determination of Relative Molecular Mass Distribution

Reverse-phase high-performance liquid chromatography (RP-HPLC) was employed to determine the relative molecular weight distribution of OPs. Briefly, 100 mg of the sample was accurately weighed into a 10 mL volumetric flask, dissolved, and diluted to the mark with the mobile phase. The mixture was ultrasonicated for 5 min, centrifuged, and the supernatant was filtered through a 0.22 μm organic phase membrane filter prior to injection. A peptide molecular weight standard set was analyzed simultaneously to establish a calibration curve for quantitative evaluation. Chromatographic separation was performed on an Agilent ZORBAX SB-C18 column (4.6 mm × 250 mm, 5 μm; Agilent Technologies, Santa Clara, CA, USA) with a mobile phase of acetonitrile/water/trifluoroacetic acid (40/60/0.1, v/v/v) under gradient elution. The column temperature was maintained at 30 °C, the detection wavelength was set at 220 nm, and the flow rate was 0.5 mL/min.

### 4.9. Identification of the Oyster Peptides

OP and DOP samples were mixed with SDT lysis buffer, and the mixture was centrifuged (14,000 rpm, 15 min) after ultrasonic extraction (30 W, 5 min). The supernatant was ultrafiltered (10 kDa) and desalinated for peptide identification.

An LC-MS/MS system (UHPLC EASY-nLC 1200 & Q Exactive HF-X, Thermo Fisher, Waltham, MA, USA) was utilized for peptide analysis. The stationary phase was a RP-C18 column (150 × 0.15 mm, Column Technology Inc., Fremont, CA, USA) and the mobile phase comprised 0.1% formic acid in water (Phase A) and 0.1% formic acid, 84% acetonitrile in water (Phase B). The gradient program was set as follows: 0–50 min, 4% B; 50–54 min, 50% B; 54–60 min, 100% B. The ion source was operated in positive electrospray ionization (ESI) modes. The sample mass charge ratio was collected according to 10 fragment maps (MS2 scan) collected after each full scan. The analysis time was 60 min.

De novo peptide sequencing was conducted via the built-in algorithm of MaxQuant 1.5.5.1 (Max Planck Institute of Biochemistry, Munich, Germany) to determine peptide amino acid sequences. Key parameters included: precursor ion mass tolerance (±10 ppm), fragment ion mass tolerance (±0.02 Da), minimum peptide length (6 residues), 0 missed cleavages, variable modifications (methionine oxidation, +15.995 Da; cysteine carbamidomethylation, +57.021 Da), and FDR ≤ 1% (for reliability). Generated sequences were validated against the UniProt database to confirm structural authenticity.

### 4.10. Cell Culture and Treatment

RAW264.7 cells were maintained in High-Glucose Dulbecco’s modified Eagle’s medium (DMEM, Procell, Wuhan, China) containing 10% fetal bovine serum, and cultured at 37 °C and 5% CO_2_. For the CCK-8 and neutral red staining test, the cells were plated in a sterile flat-bottom 96-well plate at 1000 cells/well and cultured for 24 h. For the other test, the cells were plated in a sterile flat-bottom 6-well plate with 5 × 10^6^ cells/well and cultured for 24 h.

The cells were divided into 7 groups: control group, OP treatment group (50 μg/mL, 100 μg/mL, 250 μg/mL, 500 μg/mL, and 1000 μg/mL), and positive group (1 μg/mL LPS). Three wells were repeated for each group. The intervention lasts for 24 h.

### 4.11. Cell Proliferation Detection

Cell counting kit-8 (CCK-8, Biosharp, Beijing, China) was used for cell proliferation detection according to the instructions. Briefly, the cells were added with CCK-8 (10 μL/well) and cultured for 2 h. Absorbance values were detected at 450 nm.

### 4.12. Cell Phagocytic Ability Detection

The neutral red assay kit (C0013, Beyotime, Shanghai, China) was used for cell phagocytic ability detection according to the instructions. Briefly, the cells were added with neutral red dye solution (20 μL/well), and cultured for 2 h. The dye solution was discarded, and the cracking solution was added to the plate (200 μL/well) for 10 min. Absorbance values were detected at 690 nm.

### 4.13. NO Content Detection

The supernatant of culture was collected for nitric oxide (NO) content detection using the NO content assay kit (BC1470, Solarbio, Beijing, China) according to the instructions.

### 4.14. Analysis of mRNA Expression

The total RNA of RAW264.7 cell lines was isolated using TriQuick reagent (Solarbio, Beijing, China) according to the manufacturer’s instructions. The RNA quality was confirmed by gel electrophoresis, and the concentration was detected by NanoDrop 2000 (Thermo Fisher, Waltham, MA, USA). The reverse transcription of RNA was performed using Evo M-MLV RT Mix Kit II (AG11728, Agbio, Changsha, China) according to the manufacturer’s instructions.

The primer sequences were designed using Primer 5.0 software (Table S2). All primers were synthesized by Sangon Biotech Co. (Shanghai, China). qRT-PCR (polymerase chain reaction) was performed using PerfectStart^®^ Green qPCR SuperMix (AQ601-01, TransGen, Beijing, China). β-actin was selected as the reference gene after comparative stability analysis with GAPDH using geNorm software (version 3.5), which confirmed its consistent expression across all treatment conditions.

### 4.15. Western Blot

Total protein was extracted from samples using RIPA lysis buffer supplemented with protease inhibitor, followed by incubation on ice for 5 min to achieve complete lysis. The cell lysate was then centrifuged at 12,000 rpm for 10 min at 4 °C, and the supernatant was collected. Total protein quantification was performed using the BeyoBCA Rapid Protein Assay Kit (P0398S, Beyotime, Shanghai, China), with the experimental procedure strictly adhering to the manufacturer’s instructions. An equal amount of protein was electrophoresed on a 10% sodium dodecyl sulphate-polyacrylamide gel electrophoresis (SDS-PAGE) gel at 75 V for the stacking gel and 100 V for the resolving gel. Subsequently, the protein was electrotransferred to a polyvinylidene fluoride (PVDF) membrane (Millipore, Billerica, MA, USA). The membrane was blocked with 5% BSA (dissolved in TBST) at room temperature for 1 h. The membrane was incubated with primary antibodies for 3 h at 4 °C, followed by incubation with secondary antibodies for 30 min at room temperature, and then mixed with ECL-A and ECL-B. The image was collected by a chemiluminescence imaging system (2500, Tannon, Shanghai, China).

### 4.16. Statistical Analysis

Differences among groups were analyzed using one-way analysis of variance (ANOVA) followed by the least significant difference (LSD) multiple comparison tests, at least three replicates per group. The statistical analysis and data visualization were performed by GraphPad Prism 8.0 (GraphPad Software, La Jolla, CA, USA). Statistical significance was defined as *p* < 0.05, and highly significant differences were indicated as *p* < 0.01.

## 5. Conclusions

This study innovatively prepares immunomodulatory oyster peptides (OPs) via *Lactobacillus casei* fermentation, a novel approach distinct from previous methods. Under optimized conditions, OP achieved 28.24% hydrolysis, mainly consisting of low-molecular-weight peptides (<1000 Da) with good gastrointestinal digestive stability. Notably, OP activates RAW264.7 macrophages by enhancing phagocytosis, NO production, and upregulating IL-6, IL-1β, and TNF-α mRNA expression via the MAPK pathway. These findings enable high-value utilization of oyster resources, supporting OP’s application in functional foods. Limitations include lack of in vivo validation and single-peptide activity verification. Future research should focus on animal experiments, single-peptide purification, and industrial scaling-up.

## Figures and Tables

**Table 1:** Total amino acid composition of oyster peptide (OP) and digestion oyster peptide (DOP).

Amino Acids	Contents in OP (mg/g)	Proportion of OP (%)	Contents in DOP (mg/g)	Proportion of DOP (%)
Asp	14.14	9.77	17.46	12.30
Thr	4.73	3.27	6.31	4.44
Ser	5.84	4.04	7.14	5.03
Glu	24.04	16.61	22.18	15.62
Pro	10.53	7.28	7.80	5.49
Gly	27.43	18.96	17.63	12.42
Ala	15.60	10.78	11.20	7.89
Cys	0.80	0.55	1.06	0.75
Val	5.35	3.70	7.00	4.93
Met	1.52	1.05	1.84	1.30
Ile	3.32	2.29	4.52	3.18
Leu	6.58	4.55	7.30	5.14
Tyr	2.60	1.80	4.07	2.87
Phe	4.04	2.79	4.54	3.20
Lys	10.36	7.16	10.71	7.54
His	2.76	1.91	3.50	2.47
Arg	5.06	3.50	7.72	5.44

**Table 2:** Free amino acids composition of oyster peptide (OP) and the digestion of oyster peptide (DOP).

Amino Acids	Contents in OP (mg/g)	Proportion of OP (%)	Contents in DOP (mg/g)	Proportion of DOP (%)
Asp	6.95	5.16	3.51	2.48
Thr	5.04	3.74	9.89	6.99
Ser	2.59	1.92	4.79	3.38
Glu	20.95	15.55	18.28	12.91
Pro	0.00	0	0.00	0
Gly	31.67	23.50	14.91	10.53
Ala	22.02	16.34	13.76	9.72
Cys	1.63	1.21	1.40	0.99
Val	5.61	4.16	7.02	4.96
Met	2.04	1.51	2.16	1.53
Ile	3.08	2.29	4.77	3.37
Leu	7.23	5.36	10.95	7.73
Tyr	5.65	4.19	9.96	7.03
Phe	6.45	4.79	9.80	6.92
Lys	9.45	7.01	15.00	10.59
His	1.79	1.33	3.16	2.23
Arg	2.62	1.94	12.22	8.63

**Table 3:** Results of the oyster peptide (OP) molecular weight measurement.

Molecular Weight	Peak Area Percentage (%, λ220 nm)	Number-Average Molecular Weight	Weight-Average Molecular Weight
>10,000	4.63	15,112	16,088
10,000~5000	3.10	6872	7166
5000~3000	2.33	3848	3933
3000~2000	2.00	2409	2442
2000~1000	5.84	1333	1387
1000~500	8.99	681	707
500~180	15.66	269	290
<180	57.46	38	74

**Table 4:** The amino acid sequence of the peptide segment from the oyster peptide (OP) was screened based on the PeptideRanker score and the characteristics of immune active peptides.

Order	Sequence of Peptides	Molecular Weight (Da)	Hydrophobicity (%)	PeptideRanker Score
1	PAPLWQMPKFRN	1483.7758	58.33	0.919196
2	NPVDPAPLWQMPK	1491.7544	61.54	0.892749
3	PFPL	472.2686	100	0.97648
4	VPPF	458.2529	100	0.926314
5	PLNF	489.2587	75	0.878258
6	APAPAVKYLR	1084.6393	70	0.776633
7	PPMIFKTR	988.5528	62.5	0.763725
8	PPMIFKTRIM	1232.6774	70	0.75111
9	AVNMVPFPR	1029.543	77.78	0.740246
10	FSLP	462.2478	75	0.8729
11	PFKP	487.2795	75	0.884086
12	FPSIVGRPR	1027.5927	55.56	0.763312
13	VPKPYLNHPL	1176.6655	60	0.756356
14	LPPL	438.2842	100	0.857614
15	PQDVPKKPLIMIAAR	1675.9807	66.67	0.80698
16	VPFVPISGWHGD	1309.6455	58.33	0.721777
17	IVPKPYLNHPL	1289.7496	63.64	0.691513
18	FLLK	519.3421	75	0.71686
19	SPPELPDVM	983.4634	66.67	0.691783
20	MPVTDPPLRTF	1272.6536	63.64	0.656892

## Data Availability

The original contributions presented in the study are included in the article, further inquiries can be directed to the corresponding authors.

## Supplementary Materials

The following supporting information can be downloaded at: https://www.mdpi.com/article/10.3390/md23120484/s1, Table S1: The Box–Behnken experimental design of optimization of oyster peptide preparation by *Lactobacillus casei* fermentation; Table S2: Primers of real-time fluorescence quantitative PCR; Table S3: Experimental results of response surface optimization hydrolysis; Table S4: Regression analysis results of the hydrolysis model and regression coefficients.
