# Supplementary material for: Oyster Peptides Prepared by Lactobacillus casei Fermentation Enhance Immune Activity in RAW264.7 Cells via Activation of the MAPK Pathway
Source: Mar Drugs. 2025 Dec 18;23(12):484. doi: 10.3390/md23120484 (PMC12734967; doi:10.3390/md23120484)
Supplement: Supplementary file 1 [file marinedrugs-23-00484-s001.zip › marinedrugs-4031594-supplementary.pdf]

Supplementary Materials

Table S1. The Box-Behnken experimental design of optimization of oyster peptide preparation by *Lactobacillus casei* fermentation.

Factor	Levels		
	-1	0	1
A-fermentation time (h)	16	26	36
B-inoculum amount (%)	2	4	6
C-fermentation temperature (°C)	31	37	43

Table S2. Primers of real-time fluorescence quantitative PCR.

Genes	Forward (5′ to 3′)	Reverse (5′ to 3′)
β-actin	AGGGAAATCGTGCGTGACAT	CGTTGCCAATAGTGATGACC
IL-6	GGCTAAGGACCAAGACCATCC	GCACTAGGTTTGCCGAGTAGA
IL-1β	CAAATCTCGCAGCAGCACATC	TGTCCTCATCCTGGAAGGTC
iNOS	CCGAAGCAAACATCACATTCA	GGTCTAAAGGCTCCGGGCT
TNF-α	GCTGAGGTCAATCTGCCCAA	GGGGCTCTGAGGAGTAGACA

Table S3. Experimental results of response surface optimization hydrolysis.

Order	A-fermentation time (h)	B-inoculum amount (%)	C-fermentation temperature (°C)	Y-degree of hydrolysis (%)
1	16	2	37	7.8472
2	36	2	37	14.554
3	16	6	37	10.2362
4	36	6	37	23.8057
5	16	4	31	9.4572
6	36	4	31	23.0334
7	16	4	43	8.2356
8	36	4	43	15.2728
9	26	2	31	14.076
10	26	6	31	23.2297
11	26	2	43	13.3904
12	26	6	43	20.4334
13	26	4	37	25.7249
14	26	4	37	24.7108
15	26	4	37	27.1026
16	26	4	37	26.558
17	26	4	37	27.9368

Table S4. Regression analysis results of the hydrolysis model and regression coefficients.

Source	Sum of Squares	df	Mean Square	F-value	*p*-value	Significance
Model	821.49	9	91.28	50.79	< 0.0001	**
A-fermentation time	209.00	1	209.00	116.30	< 0.0001	**
B-inoculum amount	96.87	1	96.87	53.90	0.0002	**
C-fermentation time	19.42	1	19.42	10.81	0.0134	*
AB	11.77	1	11.77	6.55	0.0376	*
AC	10.69	1	10.69	5.95	0.0448	*
BC	1.11	1	1.11	0.62	0.4570	
A^2	272.12	1	272.12	151.42	< 0.0001	**
B^2	76.29	1	76.29	42.45	0.0003	**
C^2	80.32	1	80.32	44.69	0.0003	**
Residual	12.58	7	1.80			
Lack of fit	6.39	3	2.13	1.38	0.3707	ns
Pure Error	6.19	4	1.55			
Cor Toal	834.07	16				
$R^2 = 0.9849$ Adj $R^2 = 0.9655$ Pre $R^2 = 0.8658$						

$p^* < 0.05$, $p^{**} < 0.01$
